# BET Proteins Regulate Expression of Osr1 in Early Kidney Development

**DOI:** 10.3390/biomedicines9121878

**Published:** 2021-12-10

**Authors:** Janina Schreiber, Nastassia Liaukouskaya, Lars Fuhrmann, Alexander-Thomas Hauser, Manfred Jung, Tobias B. Huber, Nicola Wanner

**Affiliations:** 1 Department of Medicine IV, Faculty of Medicine, University of Freiburg, 79106 Freiburg, Germany; j.v.schreiber@web.de; 2 III Department of Medicine, University Medical Center Hamburg-Eppendorf, 20246 Hamburg, Germany; n.liaukouskaya@uke.de (N.L.); la.fuhrmann@uke.de (L.F.); t.huber@uke.de (T.B.H.); 3 Institute of Pharmaceutical Sciences, University of Freiburg, 79104 Freiburg, Germany; alexander.hauser@pharmazie.uni-freiburg.de (A.-T.H.); manfred.jung@pharmazie.uni-freiburg.de (M.J.); 4 CIBSS—Centre for Integrative Biological Signalling Studies, University of Freiburg, 79104 Freiburg, Germany

**Keywords:** renal development, nephron number, BET proteins, epigenetic regulation, BRD4, OSR1

## Abstract

In utero renal development is subject to maternal metabolic and environmental influences affecting long-term renal function and the risk of developing chronic kidney failure and cardiovascular disease. Epigenetic processes have been implicated in the orchestration of renal development and prenatal programming of nephron number. However, the role of many epigenetic modifiers for kidney development is still unclear. Bromodomain and extra-terminal domain (BET) proteins act as histone acetylation reader molecules and promote gene transcription. BET family members Brd2, Brd3 and Brd4 are expressed in the nephrogenic zone during kidney development. Here, the effect of the BET inhibitor JQ1 on renal development is evaluated. Inhibition of BET proteins via JQ1 leads to reduced growth of metanephric kidney cultures, loss of the nephron progenitor cell population, and premature and disturbed nephron differentiation. Gene expression of key nephron progenitor transcription factor Osr1 is downregulated after 24 h BET inhibition, while Lhx1 and Pax8 expression is increased. Mining of BRD4 ChIP-seq and gene expression data identify Osr1 as a key factor regulated by BRD4-controlled gene activation. Inhibition of BRD4 by BET inhibitor JQ1 leads to downregulation of Osr1, thereby causing a disturbance in the balance of nephron progenitor cell self-renewal and premature differentiation of the nephron, which ultimately leads to kidney hypoplasia and disturbed nephron development. This raises questions about the potential teratogenic effects of BET inhibitors for embryonic development. In summary, our work highlights the role of BET proteins for prenatal programming of nephrogenesis and identifies Osr1 as a potential target of BET proteins.

## 1. Introduction

The effect of environmental influences on nephron number and consequent metabolic and epigenetic programming of nephron development has become increasingly more important in recent decades. Nephron number has been shown to influence long-term kidney function [1]. A low nephron endowment has been linked to an increased risk of developing arterial hypertension as well as chronic kidney failure [2]. Human nephron development is complete by the time of birth and no further nephron formation occurs postnatally [3]. Factors influencing nephron number are prematurity, intrauterine growth arrest, and low or high birth weight [4,5,6]. Maternal malnutrition (especially vitamin A, vitamin D, zinc, and protein deficiencies) or maternal overnutrition (obesity, hyperglycemia in poorly controlled diabetes mellitus) also correlate with a lower number of nephrons in the offspring [7]. Similarly, smoking, drug and medication exposure during pregnancy are associated with a low number of nephrons [8]. 

During kidney development, all epithelial cells of the nephron, the functional unit of the kidney, stem from the nephron progenitor cells (NPCs), which are located around the ureteric bud tips where they form the so-called cap mesenchyme in the outer cortical area of the kidney, the nephrogenic zone [9]. In a reciprocal interplay of induction with the cap mesenchyme, the ureteric bud tips continue to divide, forming the ureteric tree [10]. A subpopulation of NPCs from the cap mesenchyme aggregates and forms the developing nephron structures, while the rest of the cap mesenchyme maintains self-renewal. The continuous process of nephron formation starts as early as E13.5 and continues until day 3 after birth in mice, where nephron formation abruptly ceases due to a synchronized differentiation of NPCs [11,12]. In the cap mesenchyme, transcription factors such as Six2, Cited1, Eya1 and Osr1 define the NPC population. Upon aggregation and subsequent renal vesicle formation, the cells lose the NPC markers and upregulate genes depending on their localization within the newly forming structure, such as Pax8 and Lhx1 [13]. While transcription factor Wt1 is already present in the NPCs, it is upregulated in the proximal part of the renal vesicles and marks the (future) podocyte progenitor population, whereas Notch ligand Jagged1 (Jag1) marks the distal part of the renal vesicle [14].

Recently, the manifold role of environmental influences on nephron number and the resulting epigenetic determination of nephron development have increasingly been a focus of investigation [15,16,17,18]. The bromodomain and extra-terminal domain (BET) family is a subgroup of bromodomain-containing proteins and includes BRD2, BRD3, BRD4, and BRDT. The bromodomains bind acetylated lysines on the histone tails and trigger effector proteins to bind to the extra-terminal domain, resulting in changes in gene expression during cellular proliferation and differentiation [19]. To date, it remains unclear whether the BET proteins BRD2, BRD3, and BRD4 each exert distinct or partially overlapping functions [20]. Complete loss of one member cannot be compensated by the others [21,22,23]. Binding of BRD4 to acetylated histones has been shown to catalyze binding and consecutive activation of positive transcription elongation factor b (P-TEFb) to the C-terminal domain of BRD4 [24,25]. The active form of P-TEFb phosphorylates RNA polymerase II, thus triggering gene expression of RNA polymerase II-dependent transcription processes [25]. One of the best characterized BET inhibitors is JQ1, a triazolodiazepine. Inhibition of JQ1 is selective for all BET proteins, while binding to bromodomains of other protein families has not been shown [26]. Like the acetylated lysine of histones, the triazole ring of JQ1 forms a hydrogen bond with the asparagine in the binding pocket of the bromodomain. Thus, inhibition with JQ1 acts competitively against acetylated histones at the bromodomain-binding pocket. Through this mechanism, JQ1 prevents BET proteins from binding to chromatin, resulting in modulation of gene expression [26]. JQ1-mediated inhibition of BET proteins has previously been shown to affect cell differentiation by either promoting neural differentiation or inhibiting spermatogenesis in mice with no obvious teratogenic effects in the offspring of treated males [27,28]. Due to its short half-life [26], JQ1 is currently not used for clinical trials in humans. However, several BET inhibitors are currently in Phase I/II clinical trials (https://clinicaltrials.gov, accessed on 18 November 2021).

In the present study, the effect of BET inhibitors on renal development, renal growth, nephron progenitor self-renewal and gene expression is evaluated in order determine the role of BET proteins for renal development and prenatal renal programming.

## 2. Materials and Methods

### 2.1. Animal Handling

Mice were kept in a specific-pathogen-free environment at the Center for Experimental Models and Transgenic Service (CEMT) in Freiburg, Germany and animal research facility UKE Hamburg. All mice were raised in a 12/12 h cycle of light and darkness, with access to water and standard chow ad libitum.

### 2.2. Metanephric Kidney Culture

Timed-pregnant hNPHS2Cre [29] B6.129(Cg)-Gt(ROSA)26Sortm4(ACTB-tdTomato,-EGFP)Luo/J [30] and Tg(Six2-EGFP/cre)1Amc/J mice [31] (ages 8–16 weeks) were sacrificed at E12.5 and their embryos were harvested. The metanephroi were isolated and cultured on Transwell^®^ inserts with culture medium at the medium–air interface. Cultures were grown in DMEM medium with 10% fetal bovine serum, 100 µg/mL of penicillin, 100 µg of streptomycin and treated with inhibitors in DMSO or equivalent DMSO concentrations in the control conditions. One kidney of each embryo was used for inhibitor condition, the other for DMSO controls within each experiment. If not otherwise stated, experiments were repeated at least 3 times with 3 individual litters. 

### 2.3. Genotyping

Genotyping of the mice was performed by polymerase chain reaction (PCR) amplification of DNA isolated from tail biopsies and subsequent visualization of the amplified fragments by gel electrophoresis. The following primers were used:

Tomato/EGFP forward 5′ CTC TGC TGC CTC CTG GCT TCT 3′
Tomato/EGFP reverse wildtype 5′ CGA GGC GGA TCA CAA GCA ATA 3′
Tomato/EGFP reverse mutant 5′ TCA ATG GGC CGG GGT CGT T3′
Cre forward 5′ GCA TTA CCG GTC GAT GCA ACG AGT GAT GAG 3′
Cre reverse 5′ GAG TGA ACG AAC CTG GTC GAA ATC AGT GCG 3′

### 2.4. Whole Mount Immunofluorescence Staining

Cultured explants were fixed with cold methanol for 10 min and subsequently washed three times with room temperature PBST buffer (PBS + 0.1% Tween 20) for five minutes. Blocking solution containing 5% BSA in PBST buffer was added for three hours at room temperature. The cultures were incubated with the primary antibodies in blocking solution at 4 °C overnight. Cultures were then washed three times with blocking solution for two hours each and incubated in a 1:500 dilution of secondary antibodies and 1:1000 dilution of Hoechst nuclear dye in blocking solution overnight. The cultures were again washed three times for two hours and mounted with ProlongTM Gold Antifade mountant using a spacer. The following primary antibodies were used: rabbit anti-active Caspase 3 (1:250, AF835; R&D systems Inc., Minneapolis, MN, USA), rabbit anti JAG-1 (1:100, 2620S; Cell Signalling Technology, Danvers, MA, USA), mouse anti-Pancytokeratin (1:100, AB11213; Abcam, Cambridge, England), rabbit anti-Six2 (1:200, 11562-1-AP; Proteintech Group Inc., Rosemont, IL, USA), mouse anti-WT1 (1:100, 05-753; Merck Chemicals, Darmstadt, Germany), rabbit anti-NKCC2 (1:100, SPC-401D; StressMarq Biosciences, Cadboro Bay, BC, Canada), mouse anti E-Cadherin (1:200, 4A2C7 and 1:100, 180223; Thermo Fisher, Waltham, MA, USA), mouse anti-PCNA (1:400, PC-10, Dako, Glostrup, Denmark), LTL (1:400, FL-1321, Vector Laboratories, Burlingame, CA, USA), guinea pig anti-Nephrin (1:200, GP-N2, Progen Biotechnik GmbH, Heidelberg, Germany). All secondary antibodies from Thermo Fisher, Waltham, MA, USA. 

### 2.5. Imaging

Live imaging of cultured metanephroi from Six2.Cre;Tomato/EGFP mice was performed with a Zeiss Axio Observer after mounting the membrane inserts on a glass-bottomed dish containing 200 µL of cold PBS. Live images were taken as z-stacks with a plane distance of 10 µm. For measurements of fold growth, live images were taken on 4 consecutive days (d0 to d3) and kidney area was measured and normalized to d0 values of each individual kidney. For the glomerular counting, the z-stacked GFP Channels were orthogonally projected using Zen Blue, and analyzed with ImageJ. Stained cultures were imaged using a Zeiss Axio Observer inverted microscope or U2 LSM 510 META laser scanning microscope. 

### 2.6. ISH

Total mRNA from P1 mouse kidneys were used as a template for RT-PCR and subsequent cloning. The following primers were used:

Primer sequences for in situ hybridization.
**Brd2**Forward: CGCGGGACGCGTAGCCTTCTCTGCTGTATGAGGG Reverse: CGCGGGGCGGCCGCGAGGACTAGCTGGGGAACCAGG**Brd3**Forward: CGCGGGACGCGTCAGCCAGCAGACAGCTCA Reverse: CGCGGGGCGGCCGCTCTGTCTTCAGCCCCTGC**Brd4 (P0)**Forward: CGCGGGACGCGTCGAGGGAGGAAAGAAACAGGGG Reverse: CGCGGGGCGGCCGCTGGCTACCACTTCATGGTCAGG**Brd4 (whole mount)**Forward: CGCGGGACGCGTAAAGAAGCGCTTGGAAAACA Reverse: CGCGGGGCGGCCGCGATGCCACTGCAGCACTTTA**Cited1**Forward: CGCGGGACGCGTATGCCAACCAGGAGATGAAC Reverse: CGCGGGGCGGCCGCCAACAGAATCGGTGGCTTTT**Eya1**Forward: CGCGGGACGCGTTGCATATGGGCAAACACAGT Reverse: CGCGGGGCGGCCGCCCAGGTCCCAGATGAACACT**Lhx1**Forward: CGCGGGACGCGTCAGTGTCGCCAAAGAGAACA Reverse: CGCGGGGCGGCCGCACAAATGGTTCCCGTAGCTG**Osr1**Forward: CGCGGGACGCGTACAGAAATGGGCAGCAAAAC Reverse: CGCGGGGCGGCCGCGCGAGGCTTGGTCTTAAGTG**Pax8**Forward: CGCGGGACGCGTATACACCTCTGGGACGCAAC Reverse: CGCGGGGCGGCCGCGCTTGGCCTTGATGTAGAGC**Ret**Forward: CGCGGGACGCGTTTGGTCCAGGTCAACAACAA Reverse: CGCGGGGCGGCCGCAGATGCCGTAGCCTGCTTTA**Six2**Forward: CGCGGGACGCGTGGACCCACTGCAGCATCACC Reverse: CGCGGGGCGGCCGCTTCAGGTGCTTCTGGGGTGCAG**Wt1**Forward: CGCGGGACGCGTGCCTTCACCTTGCACTTCTC Reverse: CGCGGGGCGGCCGCGCTGAAGGGCTTTTCACTTG

PCR products were cloned into pBluescript II KS (-), linearized and transcribed with T3 and T7 RNA polymerases (Promega, Mannheim, Germany) to generate sense and antisense digoxigenin-labeled probes (digoxigenin RNA labeling mix; Roche Applied Science, Mannheim, Germany). Kidneys at postnatal day 0 were fixed overnight at 4 °C in 4% paraformaldehyde, embedded in paraffin, and sectioned at 10 μm. For mRNA detection, slides were treated with proteinase K, refixed with 4% paraformaldehyde, acetylated by using acetic anhydride (0.25% acetic anhydride in 0,1M triethanolamine (T-1377; Sigma, Schnelldorf, Germany) and hybridized at 68 °C in hybridization buffer (50% formamide, 5× SSC, yeast RNA (50 g/mL), 1% SDS, heparin (50 g/mL), 0.1% probe). Stringency washes were performed with wash I (50% formamide, 5× SSC (pH4,5), 1%SDS) and wash II (50% formamide, 2× SSC). For detection, slides were incubated with alkaline phosphatase-conjugated anti-digoxigenin antibody 1:3000 at 4 °C overnight followed by BM purple staining (Roche Applied Science, Mannheim, Germany). Digital photographs were captured on an Axioplan2 microscope (Zeiss, Oberkochen, Germany).

For whole mount in situ hybridization of kidney cultures, the tissue was fixed on Transwell filters in 4% PFA overnight. Subsequently, the kidney cultures were washed three times in PBT and then dehydrated in a descending methanol series. For hybridization, the fixed kidney cultures were rehydrated in an ascending methanol series, washed in PBT, bleached in 6% H2O2 in PBT for 1 h and treated with proteinase K for 7 min before the reaction was stopped with glycine. Subsequently, the tissue was refixed with 4% PFA containing 0.2% glutaraldehyde in PBT. Samples were prehybridized with hybridization buffer for 2 h at 68 °C before adding 5 µL per well of the AS-RNA probes and incubated for two days at 68 °C. Samples were washed 3 times each with Wash Buffer 1 and Wash Buffer 2 at 68 °C, MAB buffer and then treated with blocking solution for 90 min followed by incubation with the anti-digoxigenin antibody overnight at 4 °C. The samples were washed 15x with MAB buffer to remove non-bound antibody. Subsequently, the samples were washed repeatedly with alkaline phosphatase buffer (NTMT). BM Purple was added until clear color development was visible. The reaction was stopped simultaneously by transferring the samples in EDTA. Samples were then fixed using PFA and glutaraldehyde, washed again in PBT with EDTA, and stored briefly in this solution before imaging.

### 2.7. qPCR

mRNA was reverse transcribed to cDNA using the iScript™ cDNA synthase kit (Bio-Rad) according to the manufacturer’s instructions. qPCR was performed with Bio-Rad CFX Connect Real Time PCR Detection System in triplicate using SsoAdvanced Universal SYBR Green Supermix. The normalized ΔΔCT values were calculated in the CFX Manager program. The following Primers were used: mHprt forward 5’ GCT TTC CTT GGT CAA GCA GTA CAG 3’, mHprt reverse 5’ GAA GTG CTC ATT ATA GTC AAG GGC ATA TCC 3’ [8], Osr1 forward 5′CTGCCCAACCTGTATGGTTT, Osr1 reverse 5′ TGGCACTTTAGAAAAAGAGG [32].

### 2.8. RNA-Sequencing

BET gene expression (whole kidney): ENCODE datasets ENCSR504GEG, ENCSR537GNQ, ENCSR173PJN, ENCSR000CHA (GSE93459) were used to determine normalized read counts at E14.5, E16.5, P0 and 8w, respectively [33,34].

BET gene expression (E18.5 cap mesenchyme): Normalized read counts from wildtype control animals were used (GSE94089) [15].

Single cell sequencing data (human fetal kidney, 16 weeks) was extracted from Human fetal Kidney Atlas, Semrau lab (https://home.physics.leidenuniv.nl/~semrau/humanfetalkidneyatlas/, accessed on 4 October 2021) [35].

Kidney culture RNA-sequencing: Total RNA of kidneys grown for 24 h with DMSO or JQ1 100 nM treatment was isolated using the Qiagen RNeasy Micro Kit. RNA sequencing was performed by BGI, Hongkong and GATC, Konstanz, Germany. Quality control was carried out with FastQC (Barbraham Bioinformatics). Raw reads were trimmed using TrimGalore! (Barbraham Bioinformatics) and mapped with RNA Star to mm9 using Galaxy Freiburg. Read counts were extracted with htseq-count [36]; pair-wise differential gene expression was conducted with DESeq2 [37]. Gene set enrichment analysis was performed with Toppfun (https://toppgene.cchmc.org/, accessed on 4 October 2021). RNA-seq raw data have been deposited in NCBI’s Gene Expression Omnibus [38] and are accessible through the GEO Series accession number GSE188453 (https://www.ncbi.nlm.nih.gov/geo/query/acc.cgi?acc=GSE188453, accessed on 8 November 2021).

Osr1 FPKM values were extracted from GSE99101 (GSM2633453 and GSM2633457).

### 2.9. ChIP-Seq

Bed files were downloaded from GEO datasets: spermatids (GSE56526) [39], adipocytes (GSE59158), embryonic body (GSE76760) [40], mESC (GSE76760) [40], (GSE99101) [41]. Bed files were analyzed with ChIPseeker [42] and peaks were extracted. Annotated peaks were compared and visualized with UpsetR [43]. Gene set enrichment analysis was performed with Toppfun (https://toppgene.cchmc.org/, accessed on 10 May 2021). For visualization of peaks, GEO dataset GSE99101 (GSM2633459 and GSM2633463) were loaded into the UCSC Genome Browser. 

### 2.10. Statistics

For statistical analysis and visualization, GraphPad Prism 9 was used. For 2-way ANOVA, Dunnett’s multiple comparisons test (Figure 2B,E) or Šídák’s multiple comparisons test (Figure 3B) was used and adjusted *p*-values are given.

## 3. Results

### 3.1. BET Proteins Are Expressed in the Developing Kidney

**Figure 1:**
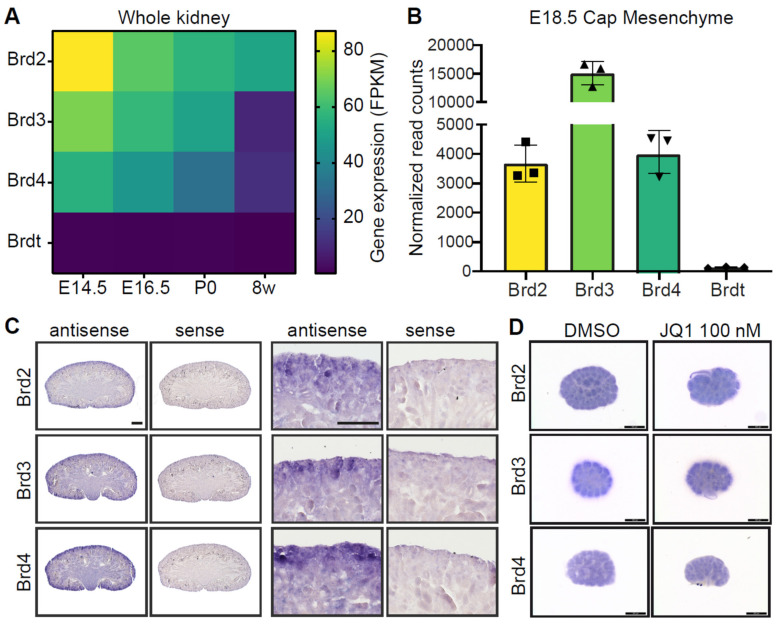
BET proteins are expressed in the developing kidney. (**A**) Heatmap of BET expression in the kidney (Encode datasets). Gene expression decreases with age. Brdt is not expressed in the kidney. FPKM, Fragments Per Kilobase of transcript per Million mapped reads. (**B**) BET proteins Brd2 (■), Brd3 (▲) and Brd4 (▼) are expressed in the cap mesenchyme at E18.5, while Brdt (♦) is not expressed. (**C**) In situ hybridization of Brd2, Brd3 and Brd4 in the P0 kidney shows gene expression in the nephrogenic zone and developing nephrons. Scale bars, 500 µm. (**D**) In situ hybridization of Brd2, Brd3 and Brd4 in E12.5 kidney cultures after 24 h in culture with DMSO or JQ1 (100 nM) treatment shows BET expression in the nephrogenic zone. Scale bar, 500 µm.

BET proteins have been described as critical for embryonic development [44]. To understand their functions in the developing kidney, ENCODE transcriptome data of the kidney at several developmental stages and adult mice were analyzed for expression of Brd2, Brd3, Brd4 and Brdt. As expected, Brdt was not expressed in the kidney (Figure 1A). Expression of Brd3 and Brd4 in the kidney was high at the developmental stages embryonic day 14.5 (E14.5), E16.5 and postnatal day 0 (P0), but was downregulated at 8 weeks. Brd2 expression was highest at E14.5 and was still present at 8 weeks (Figure 1A). Gene expression data from the isolated nephron progenitor population at E18.5 showed expression of Brd2, Brd3 and Brd4 and low counts of Brdt (Figure 1B). In situ hybridization showed enrichment of Brd2, Brd3 and Brd4 at the nephrogenic zone at P0 (Figure 1C). These results are supported by single cell sequencing data from human fetal kidneys, showing the highest expression of BRD3 in a nephron progenitor cell (NPC) subset, the pretubular aggregates (PTA) and developing nephron structures, while the highest expression of BRD2 and BRD4 was in the s-shaped bodies, distal tubules/loop of Henle, ureteric bud/collecting duct cells, PTAs and NPCs (Supplementary Figure S1). Kidney cultures of E12.5 embryonic kidneys cultured for 24 h showed BET gene expression both under control (DMSO) and BET protein inhibitory conditions (JQ1) (Figure 1D). 

### 3.2. Inhibition of BET Proteins Impairs Metanephric Kidney Development

**Figure 2:**
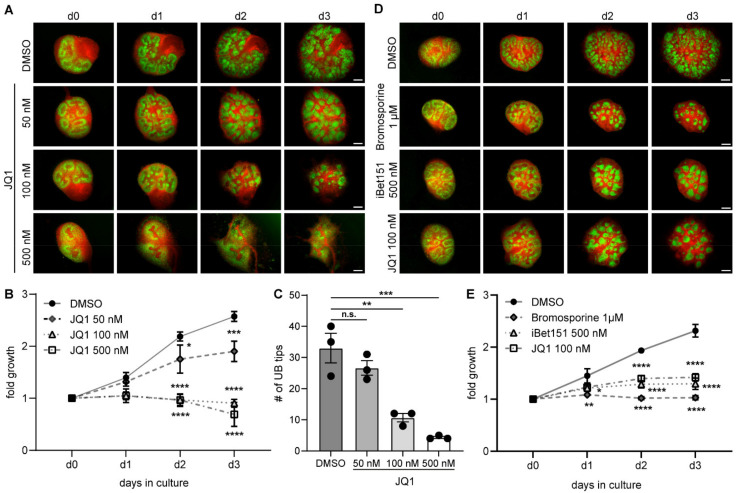
Inhibition of BET proteins impairs metanephric kidney development. (**A**) Increasing doses of BET inhibitor JQ1 (50 nM, 100 nM and 500 nM) lead to increasing changes in kidney development. Scale bars, 200 µm. (**B**) Fold growth of kidney cultures under JQ1 inhibition normalized to the individual kidney area on day 0. Two-way ANOVA multiple comparisons. * *p* < 0.05. *** *p* < 0.001. **** *p* < 0.0001. *n* = 4. (**C**) Decrease in number of ureteric bud tips at day 3 shows correlation with JQ1 concentration. One-way ANOVA multiple comparisons. n.s., not significant. ** *p* < 0.01. *** *p* < 0.001. UB, ureteric bud. *n* = 3. (**D**) Bromodomain inhibitors iBet151 (500 nM) and Bromosporine (1 µM) exhibit comparable effects to JQ1 (100 nM) on renal growth and differentiation. Scale bar, 200 µm. (**E**) Fold growth of kidney cultures under Bromosporine, iBet151 and JQ1 inhibition normalized to the individual kidney area on day 0. Two-way ANOVA multiple comparisons. * *p* < 0.05. ** *p* < 0.01. **** *p* < 0.0001. *n* = 2–3.

To understand the importance of BET proteins for the developing kidney, metanephric kidney cultures were treated with BET inhibitors. Treatment with JQ1 in increasing concentrations showed a dose-dependent effect on kidney morphology with Six2.Cre Tomato/EGFP dual fluorescent reporter mice over 3 days, leading to aggregation of EGFP-positive cells (NPCs and their offspring) at 100 nM JQ1 and halted development at 500 nM JQ1 (Figure 2A). Measurement of kidney size showed a significant reduction in fold growth after day 2 with 100 nM and 500 nM conditions (Figure 2B). Growth reduction was accompanied by decreased ureteric bud branching (Figure 2C). The phenotype was validated with different concentrations of BET inhibitors Bromosporine and iBet151, which led to similar morphological changes and reduced growth (Figure 2D–E, Supplementary Figure S2). Expression of proliferating cell nuclear antigen (PCNA) in kidney cultures at day 3 suggests continued proliferative capacity in cultures treated with 50 and 100 nM JQ1, while 500 nM halts proliferation at d3 (Supplementary Figure S3). Increased JQ1 concentration (100 nM) leads to apoptosis in stromal areas at d3 (Supplementary Figure S3).

### 3.3. BET Inhibition Leads to Depletion of Nephron Progenitor Cells

**Figure 3:**
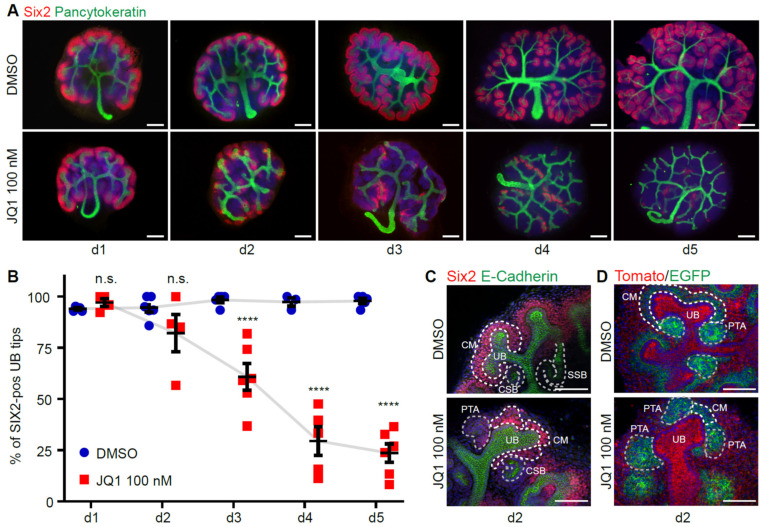
BET inhibition leads to depletion of nephron progenitor cells. (**A**) Whole mount immunofluorescence staining of SIX2 and Pancytokeratin from d1 until d5 in culture under DMSO or 100 nM JQ1 conditions. Scale bar, 200 µm. (**B**) Quantification of the ureteric bud tips surrounded by Six2-positive cap mesenchyme over time (*n* = 4–6). Two-way ANOVA multiple comparisons. n.s., not significant. **** *p*-value < 0.0001. (**C**) Morphological changes in the cap mesenchyme structure at day 2 in whole mount staining of Six2 and E-Cadherin. Scale bar, 50 µm. CM, cap mesenchyme. UB, ureteric bud. PTA, pretubular aggregate. CSB, comma-shaped body. SSB, s-shaped body. (**D**) Morphological changes in the cap mesenchyme structure at day 2 in Six2.Cre;Tomato/EGFP reporter mice. Scale bar, 50 µm.

As BET proteins were enriched in the nephrogenic zone, the NPCs were stained with marker SIX2 and imaged over time in control or JQ1-inhibitory conditions. Under control conditions, every ureteric bud tip was surrounded by SIX2-positive cap mesenchyme (Figure 3A, upper panel). However, with 100 nM JQ1 treatment, SIX2-positive populations were decreased (Figure 3A, lower panel). This decrease was visible after 2 days in culture and was significant after 3 days in culture (Figure 3B). Loss of Six2-positive progenitor cells after 3 days was also detectable with other BET inhibitors (Supplementary Figure S4). Higher magnification of the cap mesenchyme showed aggregation of the NPCs and thinning of the cortical population under BET inhibition (Figure 3C). Aggregation of NPCs, visualized by Six2.Cre;Tomato/EGFP reporter mouse lines, was partly ectopic and PTAs were enlarged (Figure 3D). 

### 3.4. BET Inhibition Leads to Abnormal Nephron Differentiation

**Figure 4:**
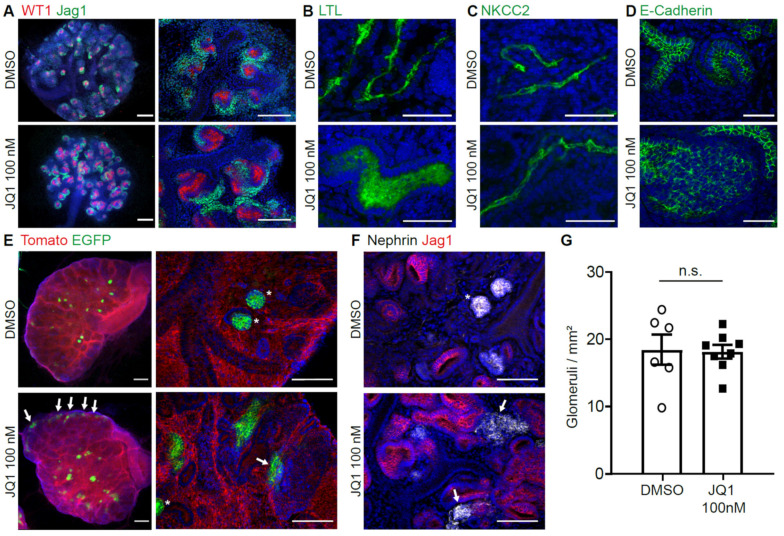
BET inhibition leads to abnormal nephron differentiation. (**A**) Whole mount immunofluorescence staining shows developing nephrons with increased size of both proximal (WT1) and distal (JAG1) part at d3 of culture. Scale bar left panel, 200 µm. Scale bar right panel, 100 µm. (**B**) Whole mount immunofluorescence staining shows dilated proximal tubules (LTL) at d5. Scale bar, 50 µm. (**C**) Thick ascending limb of loop of Henle (NKCC2) shows elongation under JQ1 inhibition at day 6. Scale bar, 50 µm. (**D**) Distal tubules (E-Cadherin) are dilated under JQ1 inhibition at day 5. Scale bar, 50 µm. (**E**) Pod.Cre;Tomato/EGFP reporter mice show development of glomeruli in kidney cultures, with ectopic signal at the capsule and deformed glomeruli under JQ1 inhibition at day 7. Asterisks, healthy glomeruli. Arrows, ectopic or deformed glomeruli. Scale bar left panel, 200 µm. Scale bar right panel, 100 µm. (**F**) Staining of podocyte marker Nephrin also shows deformed glomeruli under JQ1 inhibition at day 5 (*n* = 6–8). Asterisks, healthy glomeruli. Arrows, ectopic or deformed glomeruli. Scale bar, 100 µm. (**G**) Number of glomeruli per square mm at day 5. Unpaired *t*-test. n.s., not significant.

To visualize nephron formation upon BET inhibition, kidney cultures were stained with markers for early and mature nephron development. Upon inhibition with 100 nM JQ1, co-staining of WT1 and JAG1 showed enlargement of early nephron structures, while proximal (WT1) and distal (JAG1) compartmentalization was still maintained (Figure 4A). Proximal tubule marker Lotus Tetragonolobus Lectin (LTL) showed enlarged tubules, indicating disturbed development (Figure 4B). Loop of Henle marker NKCC2 showed thin structures expressing the marker with an increasingly elongated phenotype (Figure 4C). Distal tubule (and ureteric bud) marker E-Cadherin also showed enlarged structures (Figure 4D). Formation of mature podocytes and glomeruli was seen in Pod.Cre; Tomato/EGFP dual fluorescent reporter mice (Figure 4E). However, some of the glomeruli formed at ectopic locations and their morphology was changed with unusual elongations compared to control kidneys. This phenotype could be confirmed by stainings of podocyte marker Nephrin (Figure 4F). At day 5, the glomerular numbers measured per area were similar in control and BET inhibitory conditions (Figure 4G).

### 3.5. Loss of BET Protein Activity Leads to Downregulation of Osr1

**Figure 5:**
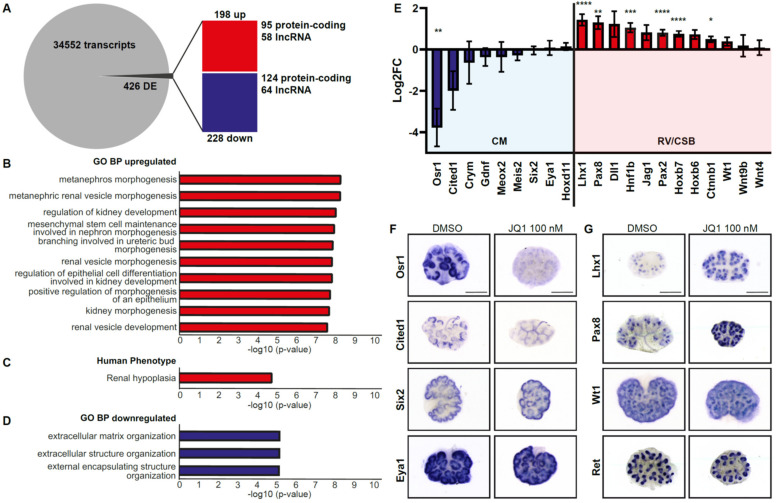
Loss of BET protein activity leads to downregulation of Osr1. (**A**) RNA-seq analysis of JQ1-treated kidney cultures shows changes in gene expression after 24 h compared to DMSO control condition (*n* = 4). DE, Differentially expressed. (**B**) Upregulated GO Biological Processes reveal prominent changes in metanephric development and renal vesicle development. (**C**) Upregulated human phenotype. (**D**) Downregulated GO Biological Processes reveal changes in extracellular matrix composition. (**E**) Key markers of the cap mesenchyme (CM), such as Osr1, are downregulated, while markers of the developing nephron (renal vesicle (RV) and comma-shaped body (CSB)) show upregulation (Lhx1, Pax8, Hnf1b, Pax2, Hoxb7). * *p* < 0.05. ** *p* < 0.01. *** *p* < 0.001. **** *p* < 0.0001. (**F**) Whole mount in situ hybridization of nephron progenitor cell-specific transcription factors confirm downregulation of Osr1 and Cited1, while Six2 and Eya1 are still expressed. Scale bar, 500 µm. (**G**) Whole mount in situ hybridization of transcription factors Lhx1 and Pax8 are upregulated, while Wt1 and Ret show no changes in expression (*n* = 3). Scale bar, 500 µm.

The marked phenotype of BET inhibition is most likely due to changes in gene expression triggered by decreased BET protein function. In order to analyze gene expression, transcriptome analysis was performed from paired cultures from the same litter with one kidney treated with DMSO and the other with JQ1 100 nM (*n* = 4 litters) for 24 h. Differential gene expression analysis showed changes in 426 genes: Upregulation of 198 (95 protein-coding) genes and downregulation of 228 (124 protein-coding) genes in the kidney culture treated with JQ1 (Figure 5A). Enrichment analysis showed upregulated GO Biological Processes such as “metanephros morphogenesis”, “metanephric renal vesicle morphogenesis” and “regulation of kidney development” (Figure 5B). Human phenotype ontology showed “renal hypoplasia” as upregulated (Figure 5C). Downregulated GO Biological Processes were “extracellular matrix organisation” and similar terms (Figure 5D). Among the cap mesenchyme markers, gene expression of Osr1 was significantly downregulated, Cited1 showed a tendency towards downregulation, while other markers such as Crym, Gdnf, Six2 and Eya1 showed no regulation (Figure 5E). Markers of the developing nephron, such as Lhx1, Pax8, Hnf1b, Pax2 and Hoxb7, were significantly upregulated, while Dll1 and Jag1 showed a tendency towards upregulation (Figure 5E). Validation of the transcriptome data by whole mount in situ hybridization on kidney cultures cultivated for 24 h confirmed loss of Osr1 and Cited1 expression in the cap mesenchyme (Figure 5F) and increase in expression in Pax8 and Lhx1 (Figure 5G) with JQ1 100 nM. Cap mesenchyme markers Six2 and Eya1, cap mesenchyme and proximal nephron marker Wt1 and ureteric bud marker Ret showed no difference in gene expression at this stage (Figure 5F,G). 

### 3.6. BRD4 Regulates Osr1 Expression 

**Figure 6:**
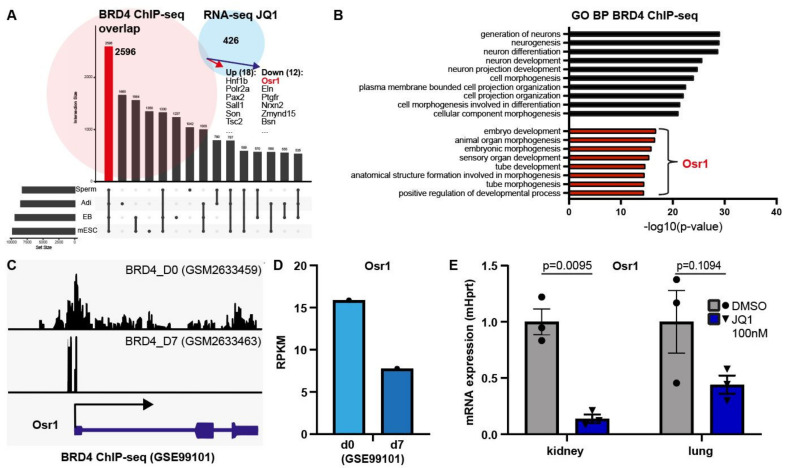
BRD4 regulates Osr1 expression. (**A**) BRD4 ChIP-seq datasets from 4 tissues during development show overlap in 2596 genes, 30 of which are also differentially regulated in the +/− JQ1-treated kidney cultures, among them Osr1 (red). Adi, Adipocytes. EB, Embryoid Body. mESC, mouse Embryonic Stem Cells. (**B**) Overlapping genes from BRD4 ChIP-seq data show overrepresentation of GO Biological Processes “generation of neurons” and “neurogenesis”(black bars), but also Osr1-regulated processes such as “embryo development” and “tube development” (red bars). (**C**) BRD4 ChIP-seq data showing high BRD4-binding at the Osr1 promotor and gene body before preadipocyte differentiation at d0 and low levels after differentiation at d7 (GSE99101). (**D**) Corresponding gene expression level of Osr1 at d0 and d7 shows correlation of gene expression with BRD4 occupancy. (**E**) qPCR of Osr1 gene expression in kidney and lung culture tissue with and without JQ1 inhibition after 24 h shows downregulation of Osr1 gene expression in both tissues after JQ1 treatment (*n* = 3). Normalized to mHprt. Unpaired *t*-test.

Of all BET proteins, BRD4 is the best characterized and has been previously studied multiple times using ChIP-sequencing. To narrow down possible targets of BRD4 in the kidney, published datasets of developing tissues from the GEO data repository were compared to determine commonly regulated genes. Out of four datasets of BRD4 ChIP-seq data from developing tissues (spermatogenesis, adipocyte differentiation, embryoid body and mouse embryonic stem cells), 2596 genes (>26% of genes) overlapped, thus highlighting genes potentially regulated by BRD4 during tissue development (Figure 6A). Out of these, 18 genes (4%) were differentially upregulated and 12 genes (3%) were downregulated in our transcriptome data (Figure 6A). From these, Osr1 stood out as a crucial gene for intermediate mesoderm development and as a cap mesenchyme marker. Enrichment analysis of the overlapping genes from the BRD4 ChIP-seq datasets showed the GO Biological Processes involved most prominently in neuron generation. GO terms in connection with the gene Osr1 were involved in embryo development and tube morphology (Figure 6B). Visualization of the ChIP-seq peak data from preadipocyte differentiation at d0 and d7 showed a strong correlation of BRD4 occupation of the promoter and gene body of the Osr1 gene with Osr1 gene expression at both stages in differentiation (Figure 6C,D), indicating higher Osr1 activation in early undifferentiated cells and lower activation in differentiated cells possibly regulated by BRD4-binding. Downregulation of Osr1 gene expression upon BET inhibition after 24 h was prominent in the kidney, but could also be shown in developing lung tissue (Figure 6E), thus pointing to Osr1 as a target of BET proteins, potentially regulated in other tissues as well.

## 4. Discussion

The number of nephrons is already prenatally determined and shows a wide interindividual range. Numerous maternal, infantile and genetic factors have been identified that are associated with a low nephron number [3,45]. Additionally, the effect of environmental and epigenetic factors on nephron number has become a focus of research in recent years [15,16,17,18]. The present work focuses on the epigenetic regulatory proteins Brd2, Brd3, and Brd4, which are expressed in the embryonic kidney and have been shown to be critical for early embryonic development [44]. However, little is known about their implications for kidney development. Expression of Brd2, Brd3 and Brd4 was detected in the nephrogenic zone, and except for Brd2, expression of BET proteins is limited to the developing tissue and decreases in the adult tissue. Because previous publications have shown that murine knock-out models of the individual BET proteins are lethal in the early embryonic period [21,22,23], we combined a pharmacological approach with metanephric organ cultures for precisely controllable conditions and ease of manipulation. All tested BET inhibitors led to growth reduction and morphological alterations in nephron structures in the metanephric cultures. JQ1 and iBet151 are selective and potent inhibitors of all BET proteins with excellent selectivity over bromodomains of other protein families [26,46,47]. Bromosporine is a pan-inhibitor of bromodomains; however, inhibition of BET proteins has been explicitly demonstrated [48,49]. With all tested BET inhibitors, loss of SIX2-positive nephron progenitor population could be shown. The aggregation and morphological shift of nephron progenitor cells towards the side of the ureteric bud and the increased differentiation of nephron anlagen suggests that pharmacological inhibition of BET proteins disrupts the self-renewal capacity of nephron progenitor cells, therefore pushing the balance towards differentiation. Decreased nephron progenitor populations affect ureteric bud branching and thus growth, as an intricate interplay between both populations is necessary for continued ureteric bud branching [50]. In our model, with the selected BET inhibitor concentration and in the measured time frame, nephron number was not affected and the number of glomeruli per area was similar in the kidney cultures with 100 nM JQ1 inhibition compared to DMSO control condition, despite the loss of the nephron progenitor population. However, for ongoing nephrogenesis in mouse and human, loss of NPCs is detrimental and early loss of NPCs would inevitably lead to (severe) kidney hypoplasia. This shows a limitation of our model, as kidney growth and nephron development can only be monitored for several days in the kidney culture system. Furthermore, the different nephron compartments showed irregularities, such as increase in size (pretubular aggregate, comma/s-shaped bodies), dilation (proximal and distal tubules), and morphological alterations (glomeruli, loop of Henle), indicating further abnormalities in gene expression downstream of the cap mesenchyme. Changes in differential gene expression with BET inhibition were confirmed by RNA-seq analysis. The ontological classification of the differentially regulated genes in RNA-seq analysis shows a phenotypic correlation to a decreased number of nephrons in mice. Due to the demonstrated early loss of the nephron progenitor cell population with consecutive differentiation, a decreased number of nephrons can be expected under BET inhibition in vivo compared to the control group. Thus, it can be assumed that BET inhibition affects key genes involved in nephron formation, kidney growth and, ultimately, nephron number. 

Gene expression analyses by RNA-seq, qPCR and whole mount in situ hybridization showed decreased expression of Osr1 after 24 h in culture with JQ1 inhibition. In the developing kidney, Osr1 expression is restricted to the nephron progenitor population with self-renewal potential [51,52]. Osr1 has been reported to play a critical role in the SIX2-dependent maintenance of the nephron progenitor cell pool, where it opposes WNT-mediated differentiation. Osr1^c/-^;Six2^GCE/+^ conditional knockout mice showed early depletion of nephron progenitor cells with loss of the entire nephrogenic zone and severe renal hypoplasia [51]. Immunofluorescence stainings of kidney cultures at E11.5 of Osr1^c/-^;Six2^GCE/+^ embryos showed loss of CITED1 and formation of large JAG1-positive structures. Thus, BET inhibition copies the renal phenotype of Osr1^c/-^;Six2^GCE/+^ embryos, highlighting Osr1 as a possible direct target gene of BET proteins during renal development. BRD4 ChIP-seq data from four developing tissues and cell lines show the Osr1 gene as a common target and indicate a correlation of Brd4 binding of the Osr1 promoter region with gene expression. These results support the hypothesis that loss of nephron progenitor cells and increased formation of WT1/JAG1-positive renal vesicles seen under BET inhibition may be due to direct regulation of Osr1 by BET proteins. While BET inhibition might affect expression of other genes as well, thereby leading to increased expression of Lhx1 and Pax8 and influencing normal nephron development, no direct link has been discovered so far. 

While this study used murine embryonic kidney cultures in an in vitro model system, the effect of BET inhibitors on the mouse and human in vivo situation is still unclear and will have to be determined in future studies. However, increased knowledge about the (epigenetic) regulation of key transcription factors of the NPCs has the potential to facilitate NPC propagation, enhance kidney organoid differentiation and impact kidney regeneration attempts by enhancing the number of NPCs and nephrons. Up to now, BET inhibitors have not been flagged as potential teratogenic agents. On the contrary, BET inhibition has even been suggested in experimental models of preeclampsia [53] or intrauterine inflammation [54]. In light of the present results, the use of BET inhibitors during fetal development should be evaluated very carefully.

## 5. Summary

The data presented in this paper show a possible direct link between epigenetic regulation of gene expression, loss of NPCs and irregular nephron formation. Early loss of NPCs leads to a low nephron number and kidney hypoplasia, and poses a major risk for chronic kidney disease later in life. More work is needed to determine possible in vivo implications for BET protein regulation in the kidney and its effect on nephron formation. Our data suggest Osr1 as a potential direct target gene of BET proteins and highlights the importance of gene regulation via BET proteins for kidney development and prenatal programming of nephron number. 

## Data Availability

RNA-seq raw data have been deposited in NCBI’s Gene Expression Omnibus [43] and are accessible through GEO Series accession number GSE188453 (https://www.ncbi.nlm.nih.gov/geo/query/acc.cgi?acc=GSE188453, accessed on 8 November 2021).

## Supplementary Materials

The following are available online at https://www.mdpi.com/article/10.3390/biomedicines9121878/s1, Figure S1. Single cell gene expression data from human fetal kidneys. Figure S2. Validation of BET inhibitors on nephrogenesis and kidney culture growth. Figure S3. Proliferation and apoptosis in the developing kidney are affected by JQ1 inhibition. Figure S4. Validation of BET inhibitors on loss of nephron progenitor cells.
