# Supplementary material for: BET Proteins Regulate Expression of Osr1 in Early Kidney Development
Source: Biomedicines. 2021 Dec 10;9(12):1878. doi: 10.3390/biomedicines9121878 (PMC8698285; doi:10.3390/biomedicines9121878)
Supplement: Supplementary file 1 [file biomedicines-09-01878-s001.zip › biomedicines-1492984-supplementary.pdf]

Supplementary Figures:

Cell type	BRD2	BRD3	BRD4
NPCa	1.839	1.79	1.668
NPCb	2.585	1.863	1.796
NPCc	2.008	1.829	1.647
NPCd	2.823	2.113	2.058
PTA	2.862	2.229	2.166
RVCSBa	2.488	2.009	1.895
RVCSBb	2.339	1.789	1.77
SSBm/d	3.261	2.034	2.526
SSBpr	2.878	1.956	2.168
SSBpod	2.247	1.868	1.815
CnT	2.716	1.702	2.13
DTLH	3.225	1.914	2.327
ErPrT	2.361	1.465	1.786
Pod	2.314	1.631	1.705
UBCD	2.984	1.719	2.312
IPC	2.616	1.897	1.917
ICa	2.142	1.598	1.642
ICb	1.778	1.476	1.476
Mes	1.655	1.482	1.818
End	2.388	1.542	1.864
Leu	2.603	1.457	1.927
Prolif	1.263	1.385	1.255

Supplementary Figure S1. Single cell gene expression data from human fetal kidneys. Data from the Human fetal Kidney atlas (PLOS biology February 21st 2019, DOI: 10.1371/journal.pbio.3000152, https://home.physics.leidenuniv.nl/~semrau/humanfetalkidneyatlas/) was mined for BET protein expression data. NPC, nephron progenitor cell. PTA, pretubular aggregate. RVCSB, renal vesicle/comma-shaped body. SSBm/d, S-shaped-body medial/distal. SSBpr, S-shaped-body proximal. SSBpod, S-shaped-body podocyte. CnT, connecting tubule. DTLH, distal tubule, loop of Henle cells. ErPrT, early proximale tubule cells. Pod, podocytes. UBCD, ureteric bud/collecting duct cells. IPC, interstitial progenitor cells. IC, interstitial cells. Mes, mesangial cells. End, endothelial cells. Leu, leukocytes. Prolif, proliferating cells.

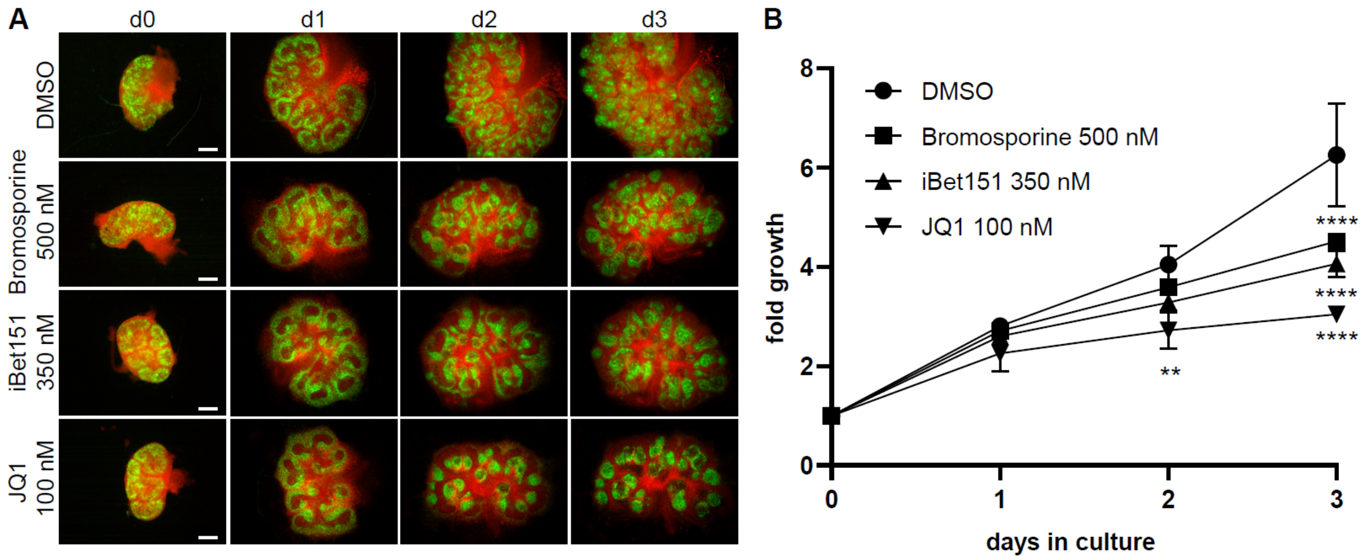

Supplementary Figure S2. Validation of BET inhibitors on nephrogenesis and kidney culture growth. A, Lower concentrations of iBET151 and Bromosporine also led to morphological changes in the Six2.Cre;Tomato/EGFP-positive kidney tissue and B, growth reduction in treated cultures. Scale bars, 200 µm. 2way ANOVA multiple comparisons.
**, p-value<0.001. ****, p-value<0.0001.

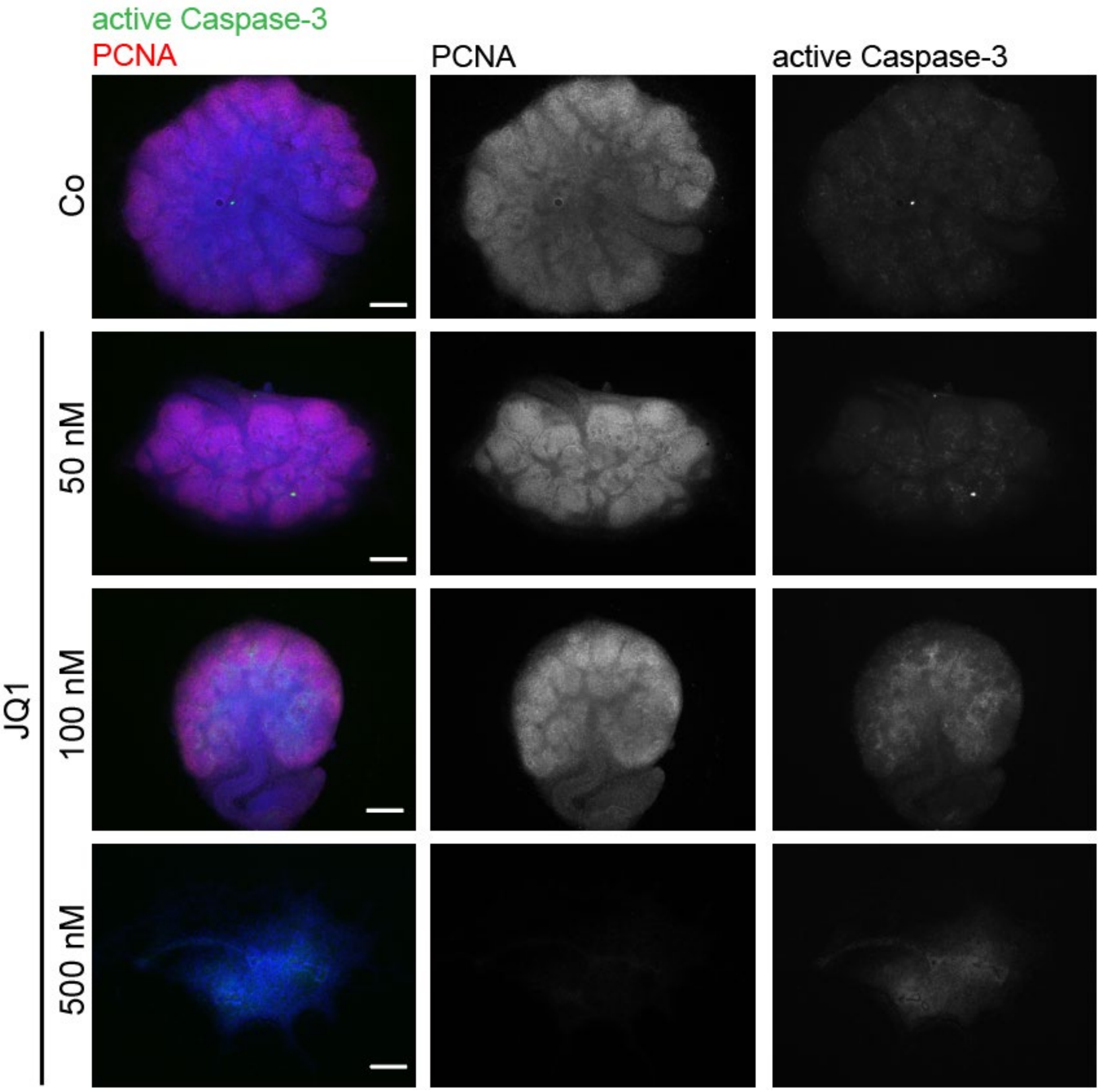

Supplementary Figure S3. Proliferation and apoptosis in the developing kidney are affected by JQ1 inhibition. Apoptosis marker active Caspase-3 and cell proliferation marker for S-phase PCNA (proliferating cell nuclear antigen) were stained in kidney cultures after three days of incubation with DMSO (control conditions), 50 nM, 100 nM or 500 nM JQ1. Scale bars, 200 µm.

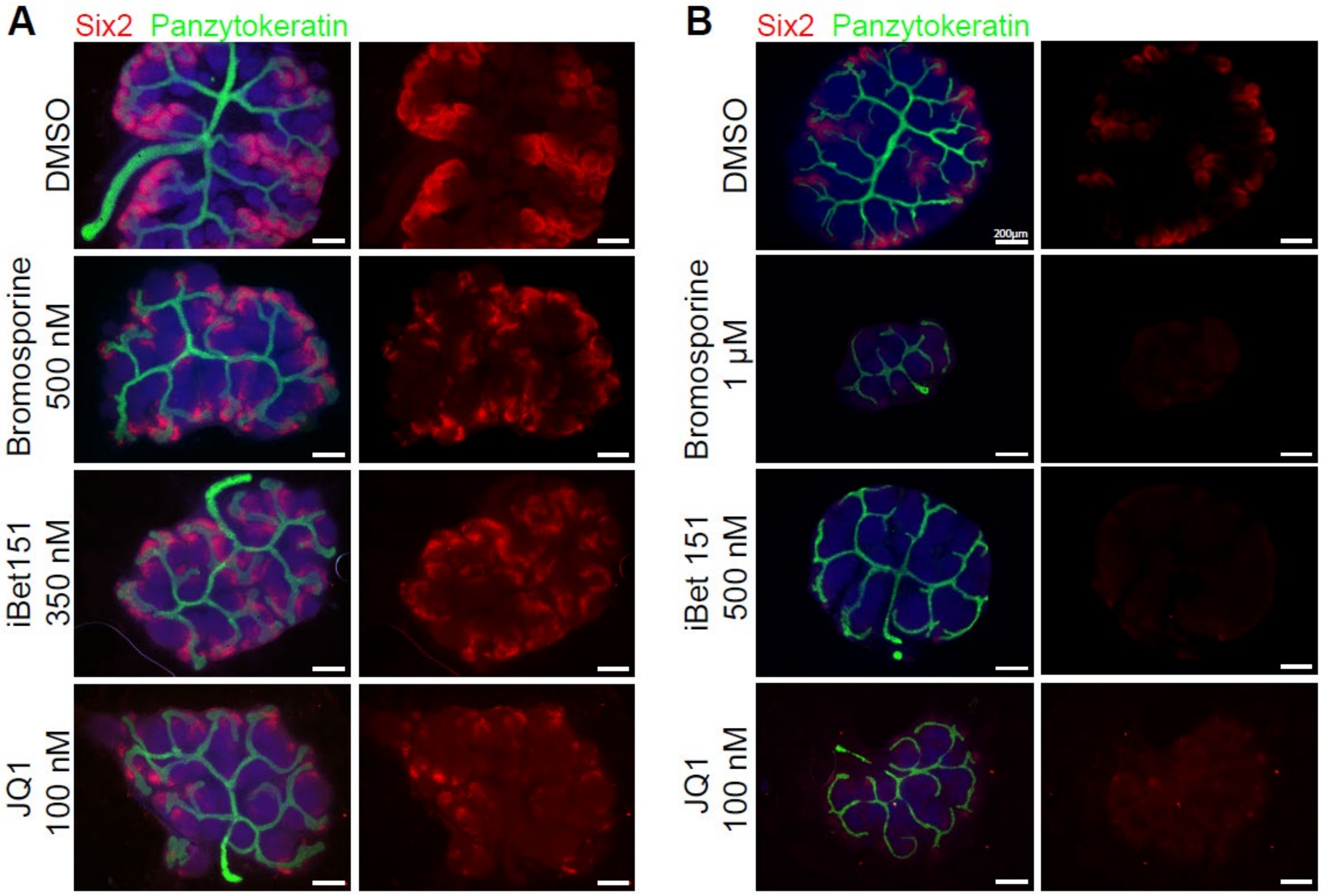

Supplementary Figure S4. Validation of BET inhibitors on loss of nephron progenitor cells. A, Lower and B, higher concentrations of Bromosporine and iBet151 showed dose-dependent effects on the progenitor population, mirroring increasing loss of Six2-positive cells with higher concentrations of iBet151 or Bromosporine a s in JQ1-treated cultures.
Day 3. Scale bars, 200 µm.
